# Clinical simulation scenarios for the planning and management of infusion therapy by nurses

**DOI:** 10.1590/0034-7167-2023-0019

**Published:** 2023-12-04

**Authors:** Mariana de Jesus Meszaros, Angélica Olivetto de Almeida, Ruana Luiz Ferreira da Silva, Aline Helena Appoloni Eduardo, Maria Helena de Melo Lima, Ana Railka de Souza Oliveira-Kumakura

**Affiliations:** I Universidade Estadual de Campinas. Campinas, São Paulo, Brazil; II Universidade Federal de São Carlos. São Carlos, São Paulo, Brazil

**Keywords:** Educational Measurement, Nursing Education, Vascular Access Devices, Simulation Training, Patient Care Planning, Evaluación Educacional, Educación en Enfermería, Dispositivos de Acceso Vascular, Entrenamiento Simulado, Planificación de Atención al Paciente, Avaliação Educacional, Educação em Enfermagem, Dispositivos de Acesso Vascular, Treinamento por Simulação, Planejamento de Assistência ao Paciente

## Abstract

**Objective:**

to build, evaluate and test two clinical simulation scenarios for the planning and management of infusion therapy by nurses.

**Methods:**

methodological study, with construction of scenarios based on the NLN Jeffries Simulation Theory and the theoretical model Vessel Health Preservation; evaluation of the scenario design by judges, with calculation of the Modified Kappa Coefficient (MKC); testing scenarios with the target audience**. Results:** scenarios built for: 1. Patient assessment and vascular device selection; and 2. Identification and management of deep vein thrombosis. In the evaluation by judges, testing of validated scenarios in relation to educational practices and simulation design, the items evaluated presented MKC values ≥ 0.74.

**Conclusion:**

two evidence-based scenarios related to infusion therapy were constructed, with high levels of agreement among judges regarding their design. In testing with nurses, good results were obtained regarding the design and structuring of educational practice.

## RESUMO

**Objetivo:**

construir, avaliar e testar dois cenários de simulação clínica para o planejamento e gerenciamento da terapia infusional pelo enfermeiro.

**Método:**

estudo metodológico, com construção de cenários baseado na *NLN Jeffries Simulation Theory* e no modelo teórico *Vessel Health Preservation*; avaliação do desenho dos cenários por juízes, com cálculo do Coeficiente de Kappa Modificado (CKM); testagem dos cenários com o público-alvo.

**Resultados:**

cenários construídos para: 1. Avaliação do paciente e seleção do dispositivo vascular; e 2. Identificação e gerenciamento de trombose venosa profunda. Na avaliação por juízes, na testagem dos cenários validados em relação às práticas educativas e ao *design* da simulação, os itens apreciados apresentaram valores do CKM ≥ 0,74.

**Conclusão:**

foram construídos dois cenários baseados em evidências relacionadas à terapia infusional, com elevados valores de concordância dos juízes, quanto ao seu desenho. Na testagem com enfermeiros, obteve-se bons resultados quanto ao desenho e estruturação da prática educativa.

## RESUMEN

**Objetivo:**

construir, evaluar y probar dos escenarios de simulación clínica para planificar y gestionar terapias de infusión por enfermeros.

**Métodos:**

es un estudio metodológico, con escenarios basados en la Teoría de la Simulación de Jeffries NLN y en el modelo teórico *Vessel Health Preservation*; evaluación del diseño de los escenarios por jueces mediante cálculo del Coeficiente de Kappa Modificado (CKM); prueba de los escenarios con el público-objeto.

**Resultados:**

escenarios construidos para: 1. evaluación de pacientes y selección de dispositivos vasculares; y 2. identificación y manejo de trombosis venosa profunda. En la evaluación por jueces, en la prueba de los escenarios validados respecto a las prácticas educativas y en el diseño de la simulación, los ítems sopesados alcanzaron el CKM ≥ 0,74.

**Conclusión:**

se construyeron dos escenarios sobre evidencias relacionadas con la terapia de infusión, con valores de concordancia altos entre los jueces, referente a su diseño. En las pruebas con enfermeros, se obtuvieron buenos resultados en cuanto al diseño y a la estructuración de la práctica educativa.

## INTRODUCTION

In recent years, the training and education of health professionals has undergone many changes with the aim of improving teaching and learning techniques^(1-3)^. In this sense, the nursing profession has made considerable advances and experienced significant changes in its knowledge, practices and functions. In view of this, the nurse needs clinical competence to properly attend and respond to these changes and advances^(4-5)^.

The concept of clinical competences in nursing goes along with the development of the profession and encompasses the combination of cognitive, psychomotor and affective skills, as described in the three major domains of Bloom’s Taxonomy^(5)^. In addition, the discussion on the development of strategies that promote patient and health professional safety has increased in recent years and has led to reflection on the teaching of professionals^(6)^.

With regard to the training of nurses, studies have identified that there are gaps and variability in nurses’ knowledge regarding infusion therapy. They also confirm the need to identify useful and adequate educational strategies to form qualified and competent teams in the insertion of vascular accesses and in the management of infusion therapy, integral parts of the professional nursing practice^(7-10)^.

Although the insertion of vascular accesses and the management of infusion therapy are basic competences for nursing professionals who are inserted in the hospital environment, they are not limited only by technical skills and require continuing education. This is because nurses need to make complex decisions daily, make clinical judgments of different levels of difficulty in order to properly assess the patient, select the most assertive vascular device, choose the best vessel for puncture and manage the entire process of infusion therapy in an efficient way. that the patient receives his therapeutic plan with safety and quality^(1-2,11)^.

In this sense, simulation training has been widely used as an innovative and effective teaching and learning strategy. It allows the development of different technical and non-technical skills that support the development of clinical judgment and favor decision making^(12-13)^.

Therefore, in view of the evidence in the literature^(7-8,11,14)^ on the insufficient and variable state of knowledge of nurses in relation to the process involving infusion therapy, the lack of training and/or continuing education that develop clinical skills related to infusion therapies and the few studies that correlate simulation training with the improvement of the skills of these professionals, it is considered opportune, the elaboration of teaching and learning strategies that may supplement these gaps.

## OBJECTIVE

To build, evaluate and test two clinical simulation scenarios for the planning and management of infusion therapy by nurses.

## METHODS

### Ethical aspects

The study was conducted in accordance with national and international ethics guidelines and approved by the Research Ethics Committee of the State University of Campinas. The Informed Consent Form was obtained from all individuals involved in the study, being signed by the expert judges online and by the target audience in writing, so that one copy was intended for the participant and the other remaining with the researchers.

### Study design, period and place

Methodological study, with a quantitative approach, following the recommendations of the Reporting guidelines for health care simulation research: extensions to the CONSORT and STROBE statements^(15)^ conducted in three steps: 1. Construction of clinical simulation scenarios and respective checklists; 2. Assessment of judge agreement; and 3. Testing the scenarios through a pilot with the target audience. The study was carried out in a university hospital and a nursing school located in the interior of São Paulo, where stages 1 and 2 took place from December 2020 to August 2021. The testing of scenarios with the target audience was carried out in October 2021.

### Study protocol

Step 1 consisted of building two high-fidelity clinical simulation scenarios and was based on the theoretical model proposed by Jeffries^(16)^, The International Nursing Association for Clinical Simulation and Learning (INACSL) guidelines)^(17)^ that conceptualize current practices to structure teaching strategies by simulation, mainly in nursing education, and include the following elements: the facilitator, the student, educational practices, simulation design (which includes objectives, fidelity, support for the participant , problem solving and debriefing) and learning outcomes. The elaboration of the debriefing phase was based on the instrument Three Stages of Efficient Debriefing Focused/Formative/Summative: a Debriefing Guide for Instructors^(18)^.

For the content of the scenario, a narrative review of the literature was carried out by the leading author in order to identify the state of the art on teaching and learning strategies and the knowledge of nurses in infusion therapy^(1-2,7-8)^, guidelines such as that of the Infusion Nurses Society (INS) are also consulted^(19)^ and the specific national guidelines of the National Health Surveillance Agency (ANVISA)^(20)^. The Vessel Health and Preservation (VHP) theoretical model^(1)^ which organizes evidence-based practices applied to infusion therapy and vascular accesses was selected for structuring the scenarios.

To examine the performance of candidates according to the skills required during the scenarios, verification checklists were prepared. This material also served to guide the debriefing stage.

In stage 2, evaluating the general structure of the scenarios and their respective checklists, the judges received an invitation to participate in the study via e-mail and after accepting and signing the Informed Consent Form (ICF), they received the file with the two scenarios and the data collection instruments, via Google Forms®. At this stage, a questionnaire was prepared for characterization and a second instrument, adapted from a previous study, which included the assessment of objectives, structure/presentation and relevance^(21-22)^ scenarios and checklists. Likert-type scales ranging from one (disagree) to four points (completely agree) were used)^(23)^. When one or two points were marked, the judges should suggest changes or deletion of the items. In addition, the judges had the opportunity to report suggestions for improvement for all constructed material^(24)^.

Step 3 consisted of testing the scenarios through a pilot with the target audience. Nurses who work in care units with adult patients at the selected hospital were invited to participate through an invitation via institutional email. After enrolling and agreeing to participate in the research, the nurses received, seven days before the application of the clinical simulation scenarios, video lessons on the VHP model^(1)^, via the Google Classroom® platform. On the day the simulation scenarios were applied, the nurses initially completed the characterization instrument. After participating in the clinical simulation, they were asked to complete the Educational Practices Questionnaire^(25)^ and the Simulation Design Scale^(26)^.

The Educational Practices Questionnaire consists of 16 items, with two subscales (one related to educational practices and another to the importance attributed to the item); this instrument is divided into four factors: 1) Active learning, 2) Collaboration, 3) Different ways of learning and 4) High expectations. The instrument uses a 5-point Likert-type scale, with the option of “not applicable” when the statement does not concern the simulated activity performed. The scale proved to be reliable, with an overall Cronbach’s alpha of 0.90^(25)^. The Simulation Design Scale is an instrument composed of 20 items, divided into two subscales (one on simulation design and the second on the importance attributed to the item); the scale is divided into five factors: 1) Objectives and information, 2) Support, 3) Problem solving, 4) Feedback and reflection and 5) Realism. The 5-point Likert-type response pattern, with the option of “not applicable” when the statement does not concern the simulated activity. The scale proved to be reliable, with an overall Cronbach’s alpha of 0.93^(26)^.

### Sample, inclusion and exclusion criteria

For the evaluation of the general structuring of the scenarios, judges who met the following criteria were invited^(27)^: a) graduation in nursing, at least 5 years of training; b) experience in infusion therapy (specialization, participation in vascular access groups or teaching in disciplines that address the theme) and/or clinical simulation, of at least 3 years.

The sample was intentional and the judges were initially selected on the Lattes Platform of the National Council for Scientific and Technological Development (CNPq) and later by the snowball selection strategy^(28)^. It was considered as an exclusion criterion: judges who did not respond within the stipulated period after accepting the invitation.

There are controversies in the literature regarding the number of judges, ranging from five to ten and other authors suggest from six to twenty judges; and in relation to the qualification of the judges, it is recommended to have clinical experience, publish and research on the subject and have experience in the conceptual structure involved^(24,28-30)^. For this study, the number of 11 responding judges was considered.

The testing of scenarios through a pilot study was carried out with a small group, selected for convenience^(24)^, being composed of eight nurses who work in the care of adult patients in infusion therapy of the selected service. The literature describes that this step plays a vital role in research so that the target population has important familiarity with the constructs through direct personal experience^(23-24,30-31)^. It was considered as an exclusion criterion: nurses who did not appear on the scheduled date or did not fill in the instruments necessary for testing the scenarios.

### Analysis of results and statistics

The results of stages 1, 2 and 3 were presented in a descriptive way and with the aid of charts and tables for better observation of the findings. The data from the judges’ evaluation and the testing of the scenarios were tabulated in an Excel® spreadsheet, and the description of the judges and nurses, respectively, was presented.

The Modified Kappa Coefficient (MKC) was calculated^(23,29,32)^, which refers to the degree of agreement between the judges, regarding the relevance, scope and comprehensibility of the items, thus guaranteeing the reliability and precision of the evaluated material. Values from 0.40 to 0.59 for the MKC are considered reasonable, from 0.60 to 0.74 good, and greater than 0.74 excellent^(33-34)^. The items that obtained MKC ≤ 0.74 underwent reformulation according to the considerations made by the judges, the consensus of the study researchers and according to the scientific evidence described in the literature.

The data collected in step 3 were compiled and analyzed using an Excel® spreadsheet, with the mean and standard deviation (SD) being calculated for each factor and item, as well as the total score.

## RESULTS

### Construction of scenarios

The construction of clinical simulation scenarios was based on the VHP model^(1)^ which structures evidence-based practices with a focus on the planning and management of infusion therapy, with two simulation scenarios being elaborated: Scenario 1 - Patient assessment and vascular device selection and Scenario 2 - Identification and management of deep vein thrombosis (DVT), with a time of 15 minutes for each scenario.

For the elaboration of the scenarios, adapted from the NLN Jeffries Simulation Theory^(35)^ four steps were considered: 1. Pre-Briefing following the Guideline and Essential Elements for Prebriefing^(36)^ which consists of presenting the simulation scenario through guidance on the simulated environment and the resources available for its realization. It is an important stage that brings security to the participants; 2. Briefing in which information about the clinical case that will be simulated is presented, that is, vignettes are made available with sufficient data about the content of the simulation^(36)^; 3. Clinical simulation scenario in which objectives, fidelity, time, problem solving, type of support for participants, script and learning outcomes were defined, exemplified in Chart 1 ^(17)^.

**Chart 1:** Summary design of clinical simulation scenarios after evaluation and testing of scenarios (N = 11). Campinas, São Paulo, Brazil, 2021

Scenarios	1. Patient assessment and vascular device selection	2. Identification and management of deep vein thrombosis
**Objectives**	**General:** Perform patient assessment for vascular device indication and selection **Specific:** a) Identify and analyze risk factors related to infusion therapy; b) Indicate the most appropriate vascular device according to the patient’s global assessment; c) Discuss with the multidisciplinary team (physician) the choice of this device.	**General:** Perform patient assessment for identification and management of suspected PICC†-related DVT^*^. **Specific:** a) Identify the risk factors for DVT; b) Evaluate the patient and identify signs/symptoms of DVT; c) Discuss the necessary conduct for the management of DVT considering the continuity of infusion therapy.
**Fidelity**	High fidelity simulation.	High fidelity simulation.
**Problem solving**	High complexity scenario in which nurses will obtain relevant information for clinical reasoning in the planning of infusion therapy and implement actions based on the association between findings in the patient’s clinical history, physical examination and therapeutic proposal.	High complexity scenario in which participants will obtain relevant information for clinical reasoning in the identification and management of DVT and implement actions based on the association between findings in the patient’s clinical history, physical examination and assessment of the vascular device.
**Cues**	a) Medical Prescription: intermittent and continuous infusions; irritant and vesicant medications; multiple infusions b) Indication of Norepinephrine infusion by the Physician; c) Provide medical records: clinical history and laboratory tests (alteration: leukocytes, lactate and platelets related to the septic condition); d) Left mastectomy; e) Hemodynamic instability.	a) Patient’s report of pain; b) Medical record with records referring to the patient’s infusion therapy during hospitalization (upper arm circumference, risk factors, PICC insertion data such as vessel diameter and number of puncture attempts); c) Medical prescription: prescription of antibiotic therapy (justifying maintenance of infusion therapy); d) Patient’s complaint: that the catheter is not good (directing the evaluation of catheter patency) and difficulty in performing some activities of daily living (for evaluation of the “Zim Zone”); e) Report of previous thrombosis. f) Intensive care unit nurse: questions the factors related to the suspicion of DVT and conduct
**Learning outcomes**	**Cognitive knowledge:** identification of the importance of the planning phase of infusion therapy for the quality of health care and patient safety. **Skill (non-technical):** development of clinical judgment to identify care needs and potential problems in order to develop actions that help solve problems and achieve favorable results for the patient in the context of infusion therapy. **Attitude:** decision-making for the indication and selection of the appropriate vascular device based on the systematic evaluation of the patient.	**Knowledge (cognitive**): identification of risk factors and signs/symptoms of DVT related to the PICC. **Skill (non-technical):** development of clinical judgment, identification of care needs and potential problems in order to develop actions that help solve problems and achieve favorable results for the patient in the management of DVT related to the PICC. **Attitude:** decision making to manage DVT related to PICC.

Note:

* DVT -Deep venous thrombosis; †PICC - Peripherally Inserted Central Catheter.

For this stage, checklists were also formulated to examine the participants’ performance and guide the subsequent debriefing stage; 4. Debriefing using the Three Stages of Efficient Focused/Formative/Summative Debriefing: A Guide to Debriefing for Instructors^(18)^ in which participants mediated by the facilitator have the opportunity to reflect on the simulated experience, the learning acquired and how much the simulation can contribute to their clinical practice.

### Content validation by judges

For the appreciation of the scenarios, 40 specialists were contacted, but each scenario was assessed by a committee formed by 11 judges who returned within the established time. These were predominantly female, with a mean age of 37 years and mean time as a nurse of 17 years, which ranged from 8 to 33 years. Three judges had a specialization, two had a master’s degree, four had a doctorate and two had a postdoctoral degree. Regarding the domain of the theme, seven judges have already taught disciplines on the theme and six judges have already participated as members of groups related to infusion therapy. Eight judges had experience in the area of clinical simulation with an average of 5 years, ranging from a minimum of 4 to a maximum of 9 years, and six judges used it as a teaching strategy in their professional practice.

Table 1 shows the MKC values for each of the evaluation items of the clinical simulation scenarios, with no item with MKC ≤ 0.74, but some adjustments suggested by the judges were made after consensus of the researchers and according to the scientific evidence described in the literature.

**Table 1:** Values of the Modified Kappa Coefficient for each of the items evaluated in the clinical simulation scenarios (N = 11). Campinas, São Paulo, Brazil, 2021

Index	Scenario 1^^*^^	Scenario 2^†^
Objectives		
Consistency of content with objectives	1.00	1.00
Clear and concise learning objectives	0.81	1.00
Content facilitates critical thinking	1.00	1.00
Problem resolution	1.00	1.00
Expected results	1.00	0.90
Objectives instigate changes in professional behavior and attitude	1.00	1.00
Structure/Presentation		
Support provided to the candidate	1.00	1.00
Target Audience	1.00	0.90
Educational Practice	1.00	1.00
Scenario fidelity	0.81	0.90
Clues	0.90	0.90
Debriefing	0.90	0.90
briefing	0.90	1.00
Case summary	1.00	0.90
Script	0.81	0.81
Materials and equipment	0.90	1.00
Check list	1.00	0.90
Scenario title	1.00	0.90
Scientifically correct information	1.00	0.90
Logical sequence of content	1.00	1.00
Information covers content on Infusion Therapy	1.00	1.00
Appropriate script for nurses	1.00	0.90
Language that is easy for the target audience to understand	1.00	1.00
Attractive view of the scenery	1.00	1.00
Data presented in a structured and objective way	1.00	1.00
Relevance		
Important content for the quality of care provided	1.00	1.00
Form of presentation contributes to the learning of nurses	1.00	1.00
Contextual details provide clues based on desired outcomes	1.00	1.00
Patient profile provides sufficient data for clinical judgment	1.00	1.00
Scenario allows the transfer of knowledge in relation to the topic	1.00	1.00
Theme portrays key aspects in relation to clinical practice	1.00	1.00
Model allows learning in different contexts	0.90	0.90
Roadmap proposes the construction of knowledge	1.00	1.00
Use by healthcare professionals and/or educators	1.00	1.00
Scenarios can circulate in the scientific community of the area	1.00	1.00
General evaluation indicators		
Adequacy of the intervention for the development of clinical judgment on the subject	1.00	1.00
Feasibility of the intervention	1.00	1.00
Adequacy of the intervention with undergraduate students	1.00	1.00
Adequacy of the intervention at the level of specialization	1.00	1.00

Note:

* Scenario 1 - Patient assessment and vascular device selection; †Scenario 2 - Identification and management of deep vein thrombosis.

In scenario 1, ‘hemodynamic instability’ was added as a clue considering the clinical picture presented in the script, the long-term bladder catheterization to the simulator for greater fidelity to the scenario and in the briefing an adjustment was necessary in the vignette since it was not clear whether the patient was being admitted or was already in bed. In scenario 2, there was a suggestion to remove the word ‘suspect’ before the term ‘deep vein thrombosis’ in the title; added in the records referring to the patient’s medical record the clues ‘vessel diameter’ and ‘number of punctures’ being correlated with Virchow’s Triad. In the script, in the DVT diagnosis stage, it was added that ‘doctor contacts via telephone for discussion regarding conduct’.

### Testing of scenarios

In testing the scenarios, 14 nurses agreed to participate in the study. However, on the date scheduled for application of the scenarios, a total of nine nurses participated, being divided into two application rounds, the first with four nurses and the second with five nurses, but one nurse was excluded from the sample for not completing the assessment instruments after the application. application of the scenarios, thus eight nurses were considered in the final sample size at this stage of the study; five nurses who previously accepted the invitation did not show up on the scheduled date. Thus, seven nurses and one male nurse made up the sample according to the inclusion criteria, with a mean age of 37 years and an average training time of 12 years, which ranged from six to 21 years. Five nurses had a specialization, two had a master’s degree and one had a doctorate. Three nurses worked in the Adult Inpatient Units, four nurses in the Adult Intensive Care Unit and one nurse in the Catheters and Infusion Therapy Group, with an average time working at the institution of ten years, ranging from six to 21 years.

Regarding updating on the subject of Vascular Access and Infusion Therapy, seven nurses participated in courses/events in the last year; all nurses are members of the catheters and infusion therapy group of their respective units in the institution and seven were trained to insert a Peripherally inserted central catheter (PICC). Regarding participation in active teaching and learning methodologies, four nurses reported having already participated in clinical simulation scenarios before. The scenarios were tested in the skills laboratory of the Faculty of Nursing at the aforementioned university, using a high-fidelity simulator (NursingAnne® by Laerdal Medical). To carry out the scenarios, two nurses with experience in clinical simulation participated as members of the simulation team, one acted as technical support, helping to structure the scenarios and acting in the role of health professionals during the simulation scenario, and the other in the simulation scenario. manipulation of audio and video equipment. The main researcher of the study played the role of facilitator in the simulation scenarios. The support team previously received the material in full with the structure of the scenarios, and the researcher standardized the content.

On the day of testing the scenarios, initially, the nurses filled out the characterization instrument, and then participated in the two clinical simulation scenarios, which were applied individually in all of its stages. In each scenario there was the participation of two volunteer nurses and the others were observers. The simulation took place in 15 minutes, and the formative debriefing took place during this same period during the practical experience and the summative debriefing in 30 minutes after the simulated experience.

The Educational Practices Questionnaire presented a total score of 4.96 (SD=0.08) in relation to the degree of agreement and 4.56 (SD=0.07) in relation to the degree of importance of the practice elements (Table 2).

**Table 2:** Scores of responses related to individual and total factors of the Educational Practices Questionnaire (n=08). Campinas, São Paulo, Brazil, 2021

Items	Degree of agreement^*^	Degree of importance^*^
	Mean^*^(SD^†^)	Mean (SD)
Factor 1 - Active learning	4.85 (0.32)	4.50 (0.10)
Factor 2 - Collaboration	5.00 (0.00)	4.63 (0.00)
Factor 3 - Different ways of learning	5.00 (0.00)	4.63 (0.00)
Factor 4 - High Expectations	5.00 (0.00)	4.50 (0.00)
Total score	4.96 (0.08)	4.56 (0.07)

Note:

* Likert scale: 1-5 - Degree of agreement: 1 - I totally disagree with the statement; 2- I disagree with the statement; 3 - Undecided-neither agree nor disagree with the statement; 4 - I agree with the statement; 5 - I totally agree with the statement; NA - Not applicable when it does not concern the simulated activity. Degree of importance: 1- Not important; 2- A little important; 3- Neutral; 4- Important; 5- Very important; †SD - Standard Deviation

For the Simulation Design Scale, Scenario 1 presented a total score of 4.73 (SD=0.07) in relation to the degree of agreement and 4.81 (SD=0.08) in relation to the degree of importance of the elements relevant to the design of the simulation; and Scenario 2 obtained a total score of 4.80 (SD=0.05) and 4.86 (SD=0.08), respectively (Table 3).

**Table 3:** Scores of the responses related to the Simulation Design Scale for Scenario 1 - Patient assessment and vascular device selection and Scenario 2 - Identification and management of deep vein thrombosis (DVT) (n=08). Campinas, São Paulo, Brazil, 2021

Items	Scenario 1		Scenario 2	
	Degree of agreement Mean^*^(SD^†^)	Degree of importance Mean (SD)	Degree of agreement Mean^*^ (SD)	Degree of importance Mean (SD)
Factor 1 - Objectives and information	4.75 (0.07)	4.80 (0.23)	4.75 (0.09)	4.80 (0.14)
Factor 2 - Support	4.81 (0.07)	4.91 (0.12)	4.81 (0.07)	4.91 (0.12)
Factor 3 - Problem Solving	4.73 (0.06)	4.75 (0.09)	4.80 (0.07)	4.93 (0.17)
Factor 4 - Feedback/Reflection	4.75 (0.00)	4.84 (0.12)	4.88 (0.00)	4.93 (0.17)
Factor 5 - Realism	4.63 (0.00)	4.75 (0.00)	4.75 (0.00)	4.75 (0.00)
Total score	4.73 (0.07)	4.81 (0.07)	4.80 (0.05)	4.86 (0.08)

Note:

* Likert scale: 1-5 - Degree of agreement: 1 - I totally disagree with the statement; 2- I disagree with the statement; 3 - Undecided-neither agree nor disagree with the statement; 4 - I agree with the statement; 5 - I totally agree with the statement; NA - Not applicable when it does not concern the simulated activity. Degree of importance: 1- Not important; 2- A little important; 3- Neutral; 4- Important; 5- Very important; †SD - Standard Deviation

## DISCUSSION

This study elaborated two clinical simulation scenarios that address the planning and management of infusion therapy by the professional nurse based on the VHP model as a theoretical reference, structured on scientific evidence that directs to the best practices in relation to the management of infusion therapy^(1)^.

The literature describes that the first stage for the construction of clinical simulation scenarios is the planning phase, which involves the diagnosis of educational needs in loco for the composition of the scenarios and the establishment of the target audience^(10,36)^. In this study, vascular access and infusion therapy gained prominence due to their relevance in the care of hospitalized patients and the fact that nurses are fundamental health professionals in this clinical scenario^(1,7,37)^.

To achieve the expected results, the development of clinical simulation scenarios must consider criteria that facilitate their effectiveness and promote solid educational experiences. The results are considered essential for learning and are correlated with the fulfillment of a set of measurable objectives as shown in Table 1. However, in order to achieve the objectives and learning outcomes, the applicability of reliable scenarios is important, therefore, the appreciation for a committee of expert judges strengthens the construction stage and the use of critical measures to assess participant performance^(10,16,36)^. Previous studies highlight that the evaluation stage by specialists is essential for the quality of the simulated practice^(29,38-39)^.

In the process of constructing the scenarios, the criteria proposed by INACLS and Jeffries’ theory were considered^(16-17)^, and, in view of this, the judges were asked about the design and presentation, with the material being considered adequate for the construction of knowledge in the context of infusion therapy, as shown in Table 1. It is worth mentioning that, despite the suitability of the scenarios for the graduation level, it should be noted that the theme must be previously adjusted for this target audience.

The simulation-based experience covers the infrastructure, people, and processes required for effective and efficient scenarios. The fidelity of the scenarios contributes to the achievement of the learning objectives, thus, the use of types of fidelity allows the necessary perception of realism for the participants to get involved in a relevant way^(40)^. Previous study^(39)^ shows that the cohesion between the types of fidelity promotes the involvement of the participant, thus, physical, conceptual and psychological fidelity were used in these scenarios, such as, for example, high fidelity simulator, patient records and active voice for the simulator, respectively .

Testing of simulation scenarios is recommended by INACSL guidelines^(24)^ in order to sustain the learning experience, identify and correct failures and allow the evaluation of the tools used. It is essential that the pilot participant is part of the target audience of the scenario^(10,24)^, corroborating with other findings in the literature^(22,38)^.

In addition, the nurses who participated in the pilot received prior educational content, through video classes, for the presentation of the VHP model, as well as prior instructions immediately before applying the scenarios. Literature recommends that pre-briefing and briefing be used to guide participants to success in the simulation-based experience^(10,36)^.

Clinical simulation scenarios should include planning for the debriefing that can be performed using different techniques, but should be guided by theoretical references and their objective is to help develop critical thinking, help in future performance and promote the integration of learning with the practice^(41)^. In this study, the Three Stages of Efficient Focused/Formative/Summative Debriefing model was chosen: a Debriefing Guide for Instructors^(18)^ for allowing a formative and summative approach and being directed to the performance of nurse educators.

Studies demonstrate that well-structured and planned clinical simulation scenarios raise the level of confidence and self-efficacy, as well as the knowledge, skills and attitudes of the participants^(38)^. The nurses evaluated the structuring of the two scenarios using the Simulation Design Scale, which were considered adequate according to the evaluated domains, as shown in Table 3. This scale includes fundamental aspects for building simulation scenarios and establishing standardized scripts and objectives, allowing that teaching and learning strategy is effective^(25,42)^. In addition, it was possible to observe from the Educational Practices Questionnaire that the nurses considered the simulation scenarios as positive teaching and learning strategies, since it was possible to visualize a high degree of agreement described in Table 2. The participants’ perception of the clinical simulation in the teaching and learning process is of paramount importance, since each subject has its own characteristics in the construction of knowledge and these particularities must be valued within the environment of educational practice^(26,43)^.

### Study limitations

It is noteworthy that the reduced number of judges in the evaluation stage and nurses in the testing of scenarios is a limiting factor of this study. The applicability of scenarios with a larger number of participants may bring more robust indicators.

### Contributions to the Nursing Area

It is important that clinical skills are learned and improved by nursing professionals in the context of infusion therapy. It is hoped that the present study will allow the use of this teaching and learning strategy in an innovative way, adding knowledge, skills and attitudes to these professionals in order to equip them for safe and quality practices.

The use of clinical simulation should be encouraged in health institutions, in order to assess the impact on the clinical judgment of nurses, as well as the use of scenarios by nurse educators in corporate, continuing and permanent education services.

## CONCLUSION

The clinical simulation scenarios entitled Scenario 1 - Patient assessment and vascular device selection and Scenario 2 - Identification and management of deep vein thrombosis (DVT) were developed based on the VHP model and guided by scientific evidence that provided the theoretical basis for its structuring. It is noteworthy that the stages of construction of simulation scenarios are essential for effective learning.

The scenarios were evaluated by judges who demonstrated high agreement regarding the structuring elements for the construction of a simulation scenario. The evaluation of the nurses in the testing stage showed good results regarding the design of scenarios and the structuring of the educational practice on aspects related to infusion therapy.

## Supplementary Material

0034-7167-reben-76-06-e20230019-suppl01

0034-7167-reben-76-06-e20230019-suppl02

## Data Availability

https://doi.org/10.25824/redu/WPENDC
