# Supplementary material for: Clinical simulation scenarios for the planning and management of infusion therapy by nurses
Source: Rev Bras Enferm. 2023 Dec 4;76(6):e20230019. doi: 10.1590/0034-7167-2023-0019 (PMC10695034; doi:10.1590/0034-7167-2023-0019)
Supplement: 0034-7167-reben-76-06-e20230019-suppl01 [file 0034-7167-reben-76-06-e20230019-suppl01.pdf]

ID	Se	Ida	Ti	Ter	Es	D	Ter	Es	Tipo	D	ex	A	Tipo_	Púb	Es	Tipo	D	m	Anos_Grupo_TerapiaInfusional
1	0	30	3	8	3	1	8	0	0	0	1	6	1.2	,2,3	1	4.5	1	0	0
2	1	49	1	20	3	0	0	1	5	1	0	0	0	0	0	0	0	1	15
3	1	46	1	23	3	0	0	0	0	1	1	5	1	3	0	0	0	1	10
4	1	40	3	18	3	0	0	1	6	0	1	6	1	3	1	4.5	1	1	4
5	1	40	1	13	0	0	0	1	2.5	1	0	0	0	0	0	0	0	1	1
6	1	37	4	13	3	0	0	0	0	0	1	4	1	,2,3	1	5	1	1	1
7	1	33	4	11	1	1	8	0	0	1	0	0	0	0	0	0	0	0	0
8	1	34	5	13	3	1	8	0	0	1	1	4	1.2	1.3	1	1.5	0	0	0
9	1	55	5	33	3	1	16	1	5	1	1	4	3	2	1	4	0	0	0
10	1	42	4	21	3	1	11	1	1	1	1	9	2	1.3	0	0	0	1	11
11	1	33	4	11	3	1	5	0	0	0	1	5	2	1	1	3	1	0	0

ID	St	Pr	Pr	Fi	Re	Pi	D	Re	In	St	Sc	M	Cl	Ti	O	O	O	As	H	As	As	O	O	A	O	O	A	D	O	O	O	O	O	Pr	O cenário de simulação clínica pode circular no meio científico da área.
1	1	1	1	0	1	0	0	1	0	1	0	1	1	0	1	1	1	1	1	1	1	1	1	1	1	1	1	1	1	1	1	1	1	1	1
2	1	1	1	1	1	1	1	1	1	1	1	1	1	1	1	0	1	1	1	1	1	1	1	1	1	1	1	1	1	1	1	1	1	1	1
3	1	1	1	1	1	1	1	1	1	1	1	1	1	1	1	1	1	1	1	1	1	1	1	1	1	1	1	1	1	1	1	1	1	1	1
4	1	1	1	1	1	1	1	1	1	1	0	0	1	1	1	1	1	1	1	1	1	1	1	1	1	1	1	1	1	1	1	1	1	1	1
5	1	1	1	1	1	1	1	1	1	1	1	1	1	1	1	1	1	1	1	1	1	1	1	1	1	1	1	1	1	1	1	1	1	1	1
6	1	1	1	1	1	1	1	1	1	1	1	1	1	1	1	0	1	1	1	1	1	1	1	1	1	1	1	1	1	1	1	1	1	1	1
7	1	1	1	1	1	1	1	1	1	1	1	1	1	1	1	1	1	1	1	1	1	1	1	1	1	1	1	1	1	1	1	1	1	1	1
8	1	1	1	0	1	1	1	1	1	1	1	1	1	1	1	1	1	1	1	1	1	1	1	1	1	1	1	1	1	1	1	1	1	1	1
9	1	1	1	1	1	1	1	1	1	1	1	1	1	1	1	1	1	1	1	1	1	1	1	1	1	1	1	1	1	1	1	0	1	1	1
10	1	1	1	1	1	1	1	1	1	1	1	1	1	1	1	1	1	1	1	1	1	1	1	1	1	1	1	1	1	1	1	1	1	1	1
11	1	1	1	1	1	1	1	1	1	1	1	1	1	1	1	1	1	1	1	1	1	1	1	1	1	1	1	1	1	1	1	1	1	1	1

ID	St	Pr	Pr	Fi	Re	Pi	D	Re	In	St	So	M	Cl	Ti	O	O	O	As	H	As	As	O	O	A	O	O	A	D	O	O	O	O	O	Pe	O cenário de simulação clínica pode circular no meio científico da área.
1	1	0	1	0	1	1	0	1	1	1	1	1	1	0	1	1	1	1	1	1	1	1	0	1	1	1	1	1	1	1	1	1	1	1	1
2	1	1	1	1	1	0	1	0	1	0	0	1	0	1	1	1	1	0	1	1	1	1	1	1	1	1	1	1	1	1	1	1	1	1	1
3	1	1	1	1	1	1	1	1	1	1	1	1	1	1	1	1	1	1	1	1	1	1	1	1	1	1	1	1	1	1	1	1	1	1	1
4	1	1	1	1	1	1	1	1	1	1	0	1	1	1	1	1	1	1	1	1	1	1	1	1	1	1	1	1	1	1	1	1	1	1	1
5	1	1	1	1	1	1	1	1	1	1	1	1	1	1	1	1	1	1	1	1	1	1	1	1	1	1	1	1	1	1	1	1	1	1	1
6	1	1	1	1	1	1	1	1	1	1	1	1	1	1	1	1	1	1	1	1	1	1	1	1	1	1	1	1	1	1	1	1	1	1	1
7	1	1	1	1	1	1	1	1	1	1	1	1	1	1	1	1	1	1	1	1	1	1	1	1	1	1	1	1	1	1	1	1	1	1	1
8	1	1	1	1	1	1	1	1	1	1	1	1	1	1	1	1	1	1	1	1	1	1	1	1	1	1	1	1	1	1	1	1	1	1	1
9	1	1	1	1	1	1	1	1	1	1	1	1	1	1	1	1	1	1	1	1	1	1	1	1	1	1	1	1	1	1	1	0	1	1	1
10	1	1	1	1	1	1	1	1	1	1	1	1	1	1	1	1	1	1	1	1	1	1	1	1	1	1	1	1	1	1	1	1	1	1	1
11	1	1	1	1	1	1	1	1	1	1	1	1	1	1	1	1	1	1	1	1	1	1	1	1	1	1	1	1	1	1	1	1	1	1	1

ID	O	O	O que você acha de utilizar como estratégia de ensino e aprendizagem estes dois cenários de simulação clínica com estudantes de graduação?
1	1	1	0
2	1	1	1
3	1	1	1
4	1	1	1
5	1	1	1
6	1	1	1
7	1	1	1
8	1	1	1
9	1	0	1
10	1	1	1
11	1	1	1

Scores das respostas relativas ao "Questionário de Práticas Educativas"		
Item (n=8)	Grau de co<br>Item<br>Média*(DF	Grau de importância<br>Item<br>Média (DP)
Fator 1) Aprendizagem ativa	4,85 (0,32)	4,50 (0,10)
1. Durante a atividade de simulação eu tive a oportunidade de discutir as ideias e os conceitos ensinados no curso com o professor e outros alunos.	5,00 (0,00)	4,50 (0,53)
2. Eu participei ativamente da sessão de debriefing após a simulação.	4,75 (0,46)	4,50 (0,76)
3. Eu tive a oportunidade de refletir mais sobre meus comentários durante a sessão de debriefing.	5,00 (0,00)	4,50 (0,53)
4. Houve oportunidade suficiente na simulação para descobrir se eu compreendi claramente o material didático.	4,00 (0,76)	4,50 (0,53)
5. Eu aprendi com os comentários feitos pelo professor antes, durante ou após a simulação.	5,00 (0,00)	4,38 (0,52)
6. Eu recebi pistas durante a simulação, em tempo oportuno.	4,75 (0,46)	4,38 (0,52)
7. Eu tive a oportunidade de discutir os objetivos da simulação com o meu professor.	5,00 (0,00)	4,38 (0,52)
8. Eu tive a oportunidade de discutir ideias e conceitos ensinados na simulação com o meu professor.	5,00 (0,00)	4,63 (0,52)
9. O professor foi capaz de responder às necessidades individuais dos alunos durante a simulação.	5,00 (0,00)	4,63 (0,52)
10. O uso de atividades de simulação tornaram meu tempo de aprendizagem mais produtivo.	5,00 (0,00)	4,63 (0,52)
Fator 2) Colaboração	5,00 (0,00)	4,63 (0,00)
11. Eu tive a oportunidade de trabalhar com meus colegas durante a simulação.	5,00 (0,00)	4,63 (0,52)
12. Durante a simulação, eu e meus colegas tivemos de trabalhar na situação clínica juntos.	5,00 (0,00)	4,63 (0,52)
Fator 3) Maneiras diferentes de aprendizagem	5,00 (0,00)	4,63 (0,00)
13. A simulação ofereceu várias maneiras para aprender o material didático.	5,00 (0,00)	4,63 (0,52)
14. Esta simulação ofereceu uma variedade de formas para avaliar a minha aprendizagem.	5,00 (0,00)	4,63 (0,52)
Fator 4) Altas expectativas	5,00 (0,00)	4,50 (0,00)
15. Os objetivos para a experiência simulada foram claros e de fácil compreensão.	5,00 (0,00)	4,50 (0,76)
16. O meu professor comunicou os objetivos e expectativas a serem alcançados durante a simulação.	5,00 (0,00)	4,50 (0,76)
Score total	4,96 (0,08)	4,56 (0,07)

*Nota (Legenda): Grau de concordância: 1 – Discordo totalmente da afirmação; 2- Discordo da afirmação; 3 – Indeciso-nem concordo e nem discordo da afirmação; 4 – Concordo com a afirmação; 5 – Concordo totalmente com a afirmação; NA – Não aplicável quando não diz respeito à atividade simulada. Grau de importância: 1- Não é importante; 2- Um pouco importante; 3- Neutro; 4- Importante; 5- Muito importante *Escala Likert: 1-5 DP: Desvio Padrão*

Scores das respostas relativas à "*Escala de Design da Simulação*" do Cenário 1 - Avaliação do paciente e seleção do dispositivo vascular e Cenário 2 - Identificação e gerenciamento da trombose venosa profunda (TVP)				
Item (n=8)	Cenário 1		Cenário 2	
	Grau de cc	Grau de ir	Grau de cc	Grau de importância
	Item	Item	Item	Item
	Média*(DF	Média (DP	Média*(DF	Média (DP)
Fator 1) Objetivos e informações	4,75 (0,07)	4,80 (0,23)	4,75 (0,09)	4,80 (0,14)
1. No início da simulação foi fornecida informação suficiente para proporcionar orientação e incentivo.	4,75 (0,46)	4,50 (0,53)	4,75 (0,43)	5,00 (0,00)
2. Eu entendi claramente a finalidade e os objetivos da simulação.	4,63 (0,52)	4,63 (0,52)	4,88 (0,33)	4,75 (0,46)
3. A simulação forneceu informação suficiente, de forma clara, para eu resolver a situação-problema.	4,75 (0,71)	5,00 (0,00)	4,63 (0,48)	4,88 (0,35)
4. Foi-me fornecida informação suficiente durante a simulação.	4,88 (0,35)	4,88 (0,35)	4,75 (0,43)	4,75 (0,46)
5. As pistas foram adequadas e direcionadas para promover a minha compreensão.	4,75 (0,46)	5,00 (0,00)	4,75 (0,43)	4,63 (0,52)
Fator 2) Apoio	4,81 (0,07)	4,91 (0,12)	4,81 (0,07)	4,91 (0,12)
6. O apoio foi oferecido em tempo oportuno.	4,75 (0,46)	4,75 (0,46)	4,75 (0,43)	5,00 (0,00)
7. A minha necessidade de ajuda foi reconhecida.	4,75 (0,46)	4,88 (0,35)	4,75 (0,43)	4,88 (0,35)
8. Eu senti-me apoiado pelo professor durante a simulação.	4,88 (0,35)	5,00 (0,00)	4,88 (0,33)	4,75 (0,46)
9. Eu fui apoiado no processo de aprendizagem.	4,88 (0,35)	5,00 (0,00)	4,88 (0,33)	5,00 (0,00)
Fator 3) Resolução de Problemas	4,73 (0,06	4,75 (0,09)	4,80 (0,07)	4,93 (0,17)
10. A resolução de problemas de forma autônoma foi facilitada.	4,63 (0,52)	4,63 (0,52)	4,88 (0,33)	5,00 (0,00)
11. Fui incentivado a explorar todas as possibilidades da simulação.	4,75 (0,46)	4,75 (0,46)	4,88 (0,33)	4,63 (0,52)
12. A simulação foi projetada para o meu nível específico de conhecimento e habilidades.	4,75 (0,46)	4,88 (0,35)	4,75 (0,43)	5,00 (0,00)
13. A simulação permitiu-me a oportunidade de priorizar as avaliações e os cuidados de enfermagem.	4,75 (0,46)	4,75 (0,46)	4,75 (0,43)	5,00 (0,00)
14. A simulação proporcionou-me uma oportunidade de estabelecer objetivos para a assistência do meu paciente.	4,75 (0,46)	4,75 (0,46)	4,75 (0,43)	5,00 (0,00)
Fator 4) Feedback/Reflexão	4,75 (0,00)	4,84 (0,12)	4,88 (0,00)	4,93 (0,17)
15. O feedback fornecido foi construtivo.	4,75 (0,46)	4,75 (0,46)	4,88 (0,33)	5,00 (0,00)
16. O feedback foi fornecido em tempo oportuno.	4,75 (0,46)	5,00 (0,00)	4,88 (0,33)	4,88 (0,35)
17. A simulação permitiu-me analisar meu próprio comportamento e ações.	4,75 (0,46)	4,75 (0,46)	4,88 (0,33)	4,75 (0,46)
18. Após a simulação houve oportunidade para obter orientação/ feedback do professor, a fim de construir conhecimento para outro nível.	4,75 (0,46)	4,75 (0,46)	4,88 (0,33)	4,88 (0,35)
Fator 5) Realismo	4,63 (0,00)	4,75 (0,00)	4,75 (0,00)	4,75 (0,00)
19. O cenário se assemelhava a uma situação da vida real.	4,75 (0,46)	4,88 (0,35)	4,75 (0,43)	4,75 (0,46)
20. Fatores, situações e variáveis da vida real foram incorporados ao cenário de simulação	4,63 (0,52)	4,75 (0,46)	4,75 (0,43)	4,75 (0,46)
Score total	4,73 (0,07)	4,81 (0,07)	4,80 (0,05)	4,86 (0,08)

*Nota (Legenda): Grau de concordância: 1 – Discordo totalmente da afirmação; 2- Discordo da afirmação; 3 – Indeciso-nem concordo e nem discordo da afirmação; 4 – Concordo com a afirmação; 5 – Concordo totalmente com a afirmação; NA – Não aplicável quando não diz respeito à atividade simulada. Grau de importância: 1- Não é importante; 2- Um pouco importante; 3- Neutro; 4- Importante; 5- Muito importante *Escala Likert: 1-5 DP: Desvio Padrão*

ID	1.	2. I	3.	4. T	5.	6. T	7. T	8.	1.	2. Se assina	3.	4.	5.	6. Você já participou de avaliação em estações de habilidades?
1	0	32	0	1	R	6	10	0	1	Cursos ou t	1	1	0	0
2	1	31	0	1	Er	2	6	1	1	Cursos ou t	1	1	0	1
3	1	34	1	1.3	Es	3	13	0	1	Cursos ou t	0	1	1	1
4	1	39	1	4	0	12	15	0	1	Cursos ou t	1	1	1	1
5	1	32	1	1.3	Te	7	8	0	1	Eventos Or	1	1	1	0
6	1	41	1	1	Cl	14	15	1	1	Cursos ou t	1	1	0	0
7	1	44	2	1	A	19	21	0	1	Congressos	1	1	1	0
8	1	40	1	1	U	15	10	1	0	Não realize	1	1	0	0
