# Supplementary material for: Clinical simulation scenarios for the planning and management of infusion therapy by nurses
Source: Rev Bras Enferm. 2023 Dec 4;76(6):e20230019. doi: 10.1590/0034-7167-2023-0019 (PMC10695034; doi:10.1590/0034-7167-2023-0019)
Supplement: 0034-7167-reben-76-06-e20230019-suppl02 [file 0034-7167-reben-76-06-e20230019-suppl02.pdf]

**INSTRUMENTO DE AVALIAÇÃO DAS ESTAÇÕES DE HABILIDADES (Avaliação Juízes Especialistas)**					
Analise cada item a seguir utilizando a escala:					
AVALIAÇÃO GERAL DO OSCE					
1	O que você acha da adequação desta intervenção (OSCE) para estimular o desenvolvimento do julgamento clínico do enfermeiro?	1	2	3	4
2	O que você acha do uso dessas estações de habilidades clínicas (OSCE) como método para avaliar o julgamento clínico do enfermeiro?	1	2	3	4
3	O que você acha da viabilidade dessa intervenção para ser aplicada com os enfermeiros, ou seja, a facilidade para implementar esta intervenção?	1	2	3	4
4	O que você acha do número de estações clínicas para avaliar o enfermeiro no planejamento e gerenciamento da terapia infusional?	1	2	3	4
5	O que você acha do tempo disponibilizado para a realização das estações clínicas?	1	2	3	4
6	O que você acha de usar esta intervenção (OSCE) com enfermeiros em nível de especialização?	1	2	3	4
7	O que você acha de usar esta intervenção (OSCE) com estudantes de graduação?	1	2	3	4
Sugestões:					
ESTAÇÃO DE HABILIDADE (número da estação) - Título					
1	O título está coerente com o conteúdo da estação de habilidade	1	2	3	4
2	O conteúdo da estação de habilidade está coerente com o objetivo da estação?	1	2	3	4
3	O(s) Objetivo(s) de aprendizagem é (são) claro(s) e conciso(s)?	1	2	3	4
4	O conteúdo da estação de habilidade facilita o pensamento crítico?	1	2	3	4
5	As informações apresentadas estão cientificamente corretas?	1	2	3	4
6	Há uma sequência lógica do conteúdo proposto?	1	2	3	4
7	As informações apresentadas na estação abrangem o conteudo proposto?	1	2	3	4
8	As informações/conteúdos abordados na estação são importantes para a qualidade da assistência prestada na prática clínica do enfermeiro?	1	2	3	4
9	O objetivo da estação instiga/convida a mudanças de comportamento e atitude dos profissionais enfermeiros?	1	2	3	4
10	A linguagem utilizada na estação de habilidade é de fácil compreensão para os enfermeiros?	1	2	3	4
11	A vinheta está coerente com o conteúdo da estação de habilidade?	1	2	3	4
12	A forma de apresentação da estação de habilidade contribui para o aprendizado dos enfermeiros?	1	2	3	4
13	O checklist de avaliação está coerente com o conteúdo da estação de habilidade?	1	2	3	4
14	O roteiro para o ator está coerente com o conteúdo da estação clínica?	1	2	3	4
15	Os recursos necessários estão coerentes com o conteúdo da estação de habilidade?	1	2	3	4
Sugestões:					

INSTRUMENTO DE AVALIAÇÃO DAS ESTAÇÕES DE HABILIDADES (Testagem dos cenários)					
Analise cada item a seguir utilizando a escala:<br>1 - Discordo totalmente<br>2 - Discordo parcialmente<br>3 - Concordo parcialmente<br>4 - Concordo totalmente					
AVALIAÇÃO GERAL DO OSCE					
1	O que você achou da adequação desta intervenção (OSCE - estações clínicas) para estimular o desenvolvimento do seu julgamento clínico?	1	2	3	4
2	O que você achou do uso dessas estações de habilidades clínicas (OSCE) como método para avaliar o seu julgamento clínico?	1	2	3	4
3	O que você achou da viabilidade dessa intervenção (OSCE) para ser aplicada com os enfermeiros, ou seja, a facilidade para implementar esta intervenção?	1	2	3	4
4	O que você achou do número de estações clínicas para lhe avaliar no planejamento e gerenciamento da terapia infusional?	1	2	3	4
5	O que você achou do tempo disponibilizado para a realização das estações clínicas?	1	2	3	4
6	O que você acha de usar esta intervenção (OSCE) com enfermeiros em nível de especialização?	1	2	3	4
7	O que você acha de usar esta intervenção (OSCE) com estudantes de graduação?	1	2	3	4
8	O conteúdo das estações de habilidades facilitou o seu pensamento crítico?	1	2	3	4
9	Há uma sequência lógica do conteúdo proposto?	1	2	3	4
10	As informações apresentadas nas estações abrangem o conteúdo proposto?	1	2	3	4
11	As informações/conteúdos abordados nas estações são importantes para a qualidade da assistência prestada na sua prática clínica?	1	2	3	4
12	Os objetivos das estações instigam/convidam a mudanças de comportamento e atitude dos profissionais enfermeiros?	1	2	3	4
13	A linguagem utilizada nas estações de habilidades é de fácil compreensão?	1	2	3	4
14	A forma de apresentação das estações de habilidades contribuiu para o seu aprendizado?	1	2	3	4
15	O conteúdo das estações de habilidades facilitou a construção do seu conhecimento?	1	2	3	4
Sugestões:					
